# Accuracy of Clinical Staging of Localized Colon Cancer: A National Cancer Database Cohort Analysis

**DOI:** 10.1245/s10434-024-15875-9

**Published:** 2024-07-29

**Authors:** Sameh Hany Emile, Nir Horesh, Zoe Garoufalia, Justin Dourado, Peter Rogers, Ebram Salama, Steven D Wexner

**Affiliations:** 1 Ellen Leifer Shulman and Steven Shulman Digestive Disease Center, Cleveland Clinic Florida, Weston, FL USA; 2 General Surgery Department, Colorectal Surgery Unit, Mansoura University Hospitals, Mansoura, Egypt; 3 Department of Surgery and transplantation, Sheba Medical Center, Ramat-Gan, Israel

## Abstract

**Background:**

This study aimed to assess concordance between clinical and pathologic assessment of colon cancer.

**Patients and Methods:**

A retrospective cohort analysis of patients with stage I–III colon cancer in the National Cancer Database (2010–2019) was conducted. Concordance between clinical and pathologic assessment of colon cancer was calculated using Kappa coefficients and 95% confidence intervals (CIs).

**Results:**

A total of 125,473 patients (51.2% female; mean age 68.2 years) were included. There was moderate concordance between clinical and pathologic T stage (Kappa = 0.606, 95%CI: 0.602–0.609) and between clinical and pathologic N stage (Kappa = 0.506, 95%CI: 0.501–0.511). For right-sided colon cancer, there was moderate agreement between clinical and pathologic T stage (Kappa = 0.594, 95%CI: 0.589–0.599) and N stage (Kappa = 0.530, 95%CI: 0.523–0.537). For left-sided colon cancer, there was substantial agreement between clinical and pathologic T stage (Kappa = 0.624, 95%CI: 0.619–0.630) and moderate agreement between N stage (Kappa 0.472, 95%CI: 0.463–0.480). Sensitivity of clinical assessment of T and N stage ranged from 64.3% to 77.2% and 41.6% to 54.5%, respectively. Specificity ranged from 96.7% to 97.7% for T stage and 95.7% to 97.3% for N stage.

**Conclusions:**

Clinical assessment of T and N stages of colon cancer had good diagnostic accuracy with moderate concordance with the final pathologic stage. While clinical assessment was highly specific with < 3% of patients being over-staged, it had modest sensitivity, especially for detection of nodal involvement. Diagnostic accuracy of clinical assessment of right and left colon cancers was similar, except for higher sensitivity and accuracy of assessment of nodal involvement in right than left colon cancers.

**Supplementary Information:**

The online version contains supplementary material available at 10.1245/s10434-024-15875-9.

Colorectal cancer (CRC) is one of the most common cancers and is responsible for a considerable portion of cancer-related mortality.^1^ The prognosis of colon cancer is mainly stage-dependent. The 5-year relative survival rate of early, localized colon cancer may reach up to 91%; however, it declines to 72% in regional disease and 13% in patients with distant metastases.^2^ Careful and detailed assessment of colon cancer at presentation is crucial for planning treatment since treatment of metastatic disease is different from that of locally advanced disease with no distant spread.^3^ After confirming the diagnosis of colon cancer with colonoscopy and histologic examination of tissue biopsies, preoperative assessments aim to determine the TNM stage of the disease.^4^ Toward this end, different imaging modalities are used, including abdominopelvic computed tomography (CT) scanning, endoscopic ultrasound, magnetic resonance imaging (MRI), and positron emission tomography (PET)-CT scanning. The type and modality of tests used for preoperative assessment of colon cancers can be different from those for rectal cancers. While MRI is the standard of care for preoperative assessment of rectal cancer,^5^ CT scanning with intravenous contrast or MRI may be used for the preoperative assessment of colon cancer.^6^

In our previous study from the National Cancer Database (NCDB),^7^ we found a fair-to-moderate agreement between the clinical and pathologic assessment of the T and N stages of rectal cancer. However, because of certain anatomic considerations and differences in the assessment modalities, the accuracy of clinical assessment of colon cancer may not be similar to that of rectal cancer. A meta-analysis of the diagnostic accuracy of CT scanning in the clinical staging of colon cancer showed an excellent sensitivity in the detection of T3–T4 tumors (90%). However, the sensitivity in the detection of nodal involvement was 71%.^8^ We conducted the present study to assess the accuracy of clinical assessment of colon cancer by examining the concordance between the clinical and pathologic T and N stages, using the NCDB that we used in our previous analysis. We hypothesized that the accuracy of clinical assessment of colon cancer, using data from a large national database, may be different from that in the former meta-analysis and we also assumed that the accuracy of the assessment may vary according to the tumor location and sidedness.

## Patients and Methods

### Study Design and Setting

We conducted a retrospective cohort study on patients with clinical stage I–III colon cancer who underwent colectomy. Data from the NCDB between 2010 and 2019 were used to assess the agreement between the clinical and final pathologic assessment of the T and N stages of colon cancer. Data collected from > 1500 Commission on Cancer (CoC)-accredited hospitals across the USA are included in the NCDB, which is a joint project of the CoC of the American College of Surgeons and the American Cancer Society. “The de-identified data used in the study are derived from the NCDB and its participating hospitals that are not responsible for the statistical validity of the analysis or the conclusions of the study.” Approval from the ethics committee and written consent to participate in the study were not required owing to the retrospective nature of the study, which involved a review of a national database that included de-identified data.

### Study Population

The inclusion criteria for the study were patients with clinical stage I–III colonic adenocarcinoma (ICDO-3 code 8140/3, 8480-8481/3, 8490/3) who underwent colectomy without any preoperative treatment. Patients were selected using the NCDB variable for clinical stage summary American Joint Commission on Cancer (AJCC) Clinical Stage Group, which indicates the overall TNM stage of colon cancer according to the 7th and 8th editions of the TNM system. For the purpose of this study, stages IA, IB, and IC were grouped as stage I and the same for stages II A, B, and C, and stages III A, B, and C. We excluded patients with other histologic types of colon cancer, patients with stage IV disease or unknown clinical stage, patients who did not undergo surgery, underwent local excision, or had a nonspecified procedure, and patients who received neoadjuvant therapy to avoid the downstaging effect of neoadjuvant therapy when assessing the concordance between clinical and pathologic stages.

### Data Collection

The following datapoints were included in the analysis: age, sex, race, Charlson comorbidity index score, insurance status, residence area, clinical and pathologic TNM stage, tumor location, type of surgery, and surgical approach.

### Outcomes

The main outcome of the study was the agreement between the clinical and pathologic assessment of T and N stage of colon cancer, stratified by tumor location. Other outcomes included the diagnostic accuracy of the clinical assessment of the TN stage of colon cancer. Under-staging was defined as having a lower clinical stage than the final pathologic stage (e.g., cT1 and pT3), whereas over-staging was defined as having a higher clinical stage than the final pathologic stage (e.g., cT4 and pT2).

### Statistical Analysis

Statistical analyses were performed using EZR (version 1.55) and R software (version 4.1.2). Continuous data were expressed in the form of mean and standard deviation and were processed using the Student’s *t* test. Categorical data were expressed as numbers and absolute proportions and were analyzed with the Fisher exact test or Chi-Square test. The concordance between the clinical and pathologic assessment of the T and N stages of colon cancer was assessed using the Kappa coefficient and its 95% confidence interval (CI). A Kappa < 0 indicated no agreement, 0–2 indicated slight agreement, 0.21–0.4 indicated fair agreement, 0.41–0.6 indicated moderate agreement, 0.61–0.8 indicated substantial agreement, and 0.81–1 indicated an almost perfect agreement as Landis and Koch suggested.^9^ *P*-values < 0.05 were considered significant.

The diagnostic accuracy of clinical assessment was summarized as sensitivity, specificity, and accuracy on the basis of the numbers of true and false positives and true and false negatives. The diagnostic accuracy parameters were calculated using MedCalc Software Ltd. Diagnostic test evaluation calculator. (www.medcalc.org/calc/diagnostic_test.php). The sensitivity of clinical assessment of T stage was based on the distinction between early (T1–2) and advanced (T3–4) disease, following the concept adopted in a previous meta-analysis on CT staging of colon cancer.^8^ The sensitivity of clinical assessment of N stage was based on the distinction between absent nodal disease (N0) and positive nodal disease (N1–2). True positives were patients with positive findings (T3–4 or N1–2) in both clinical and pathologic assessments, false positives were patients with positive findings in clinical assessment and negative findings in pathology (cT3–4 versus pT1–2 or cN1–2 versus pN0), and false negatives were patients with negative clinical findings and positive pathology findings (cT1–2 versus pT3–4 or cN0 versus pN1–2).

## Results

### Cohort Description

**Fig. 1:**
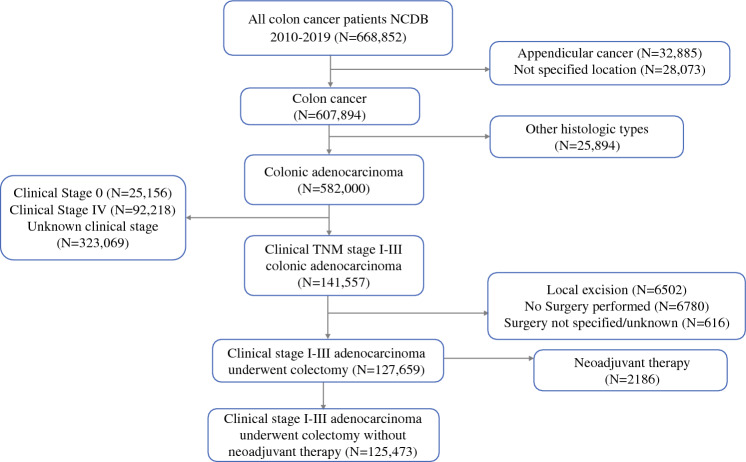
Flow chart for patient inclusion in the study

After screening the records of 582,000 patients with colonic adenocarcinoma, 125,473 (51.2% female) were included in the study (Fig. 1). The mean age of patients was 68.2 ± 13.3 years. Most patients were White (83%), had a Charlson score < 2 (89.7%), were Medicare-insured (57.1%), and lived in a metropolitan area (85.6%). Overall, 47.3% of patients had clinical stage I, 32% had stage II, and 20.7% had stage III disease. Most procedures were performed via a minimally invasive approach.

### Analysis of the Entire Cohort

**Fig. 2:**
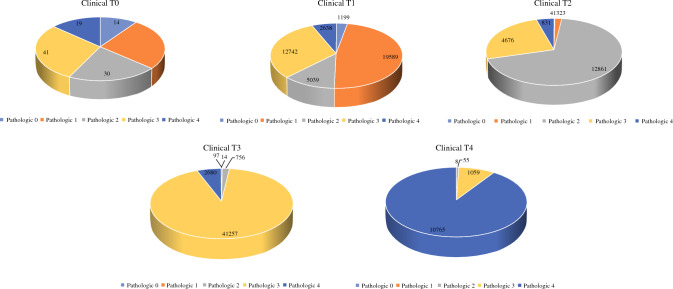
Pie chart showing the final pathologic stage for each clinical T stage

**Fig. 3:**
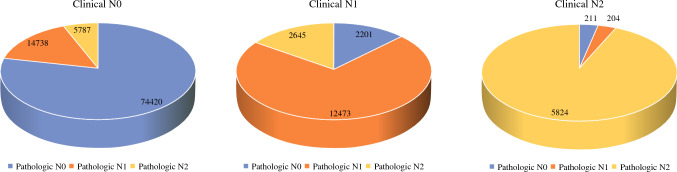
Pie chart showing the final pathologic stage for each clinical N stage

After the exclusion of patients with Tx and Nx stage, there was a moderate concordance between the clinical and pathologic assessment of colon cancer for the T stage [Kappa = 0.606, 95%CI: 0.602–0.609, standard error (SE) = 0.002], N stage (Kappa = 0.506, 95%CI: 0.501–0.511, SE = 0.002; Figs. 2,3), and TNM stage (Kappa = 0.582, 95%CI: 0.578–0.586).

The sensitivity of clinical assessment in distinguishing early T stage (T1–2) from advanced T stage (T3–T4) was 72.7% (95%CI: 72.4–73%), specificity was 97.7% (95%CI: 97.5–97.8%), accuracy was 81.3% (95%CI: 81–81.5%), positive predictive value (PPV) was 98.3% (95%CI: 98.2–98.4), and NPV was 64.4% (95%CI: 64.1–64.7%). The sensitivity of clinical assessment in the detection of nodal disease (N1–2 stage) was 50.7% (95%CI: 50.3–51.2%), specificity was 96.9% (95%CI: 96.7–96.9%), accuracy was 80.6% (95%CI: 80.4–80.9%), PPV was 89.8% (95%CI: 89.4–90.1), and NPV was 78.4% (95%CI: 78.2–78.5%).

### Analysis of Right-Sided Colon Cancer

There was moderate agreement between clinical and pathologic assessment of right colon cancer for T stage (Kappa = 0.594, 95%CI: 0.589–0.599, SE = 0.003), N stage (Kappa = 0.530, 95%CI: 0.523–0.537, SE = 0.003), and TNM stage (Kappa = 0.591, 95%CI: 0.586–0.596, SE = 0.003).

The sensitivity of clinical assessment in distinguishing early T stage (T1–2) from advanced T stage (T3–T4) was 72.3% (95%CI: 71.8–72.7%), specificity was 97.5% (95%CI: 97.3–97.7%) accuracy was 80.4% (95%CI: 80.1–80.7%), PPV was 98.4% (95%CI: 98.2–98.5), and NPV was 62.3% (95%CI: 61.9–62.7%). The sensitivity of clinical assessment in the detection of nodal disease (N1–2 stage) was 54.5% (95%CI: 53.8–55.2%), specificity was 96.5% (95%CI: 96.3–96.7%), accuracy was 81.9% (95%CI: 81.5–82.2%), PPV was 89.3% (95%CI: 88.8–89.8%), and NPV was 79.8% (95%CI: 79.6–80.1%).

### Analysis of Left-Sided Colon Cancer

There was substantial agreement between the clinical and pathologic assessment for the T stage of left colon cancer (Kappa = 0.624, 95%CI: 0.619–0.630, SE = 0.003) and a moderate agreement for the N stage (Kappa = 0.472, 95%CI: 0.463–0.480, SE = 0.004) and TNM stage (Kappa = 0.572, 95%CI: 0.566–0.578, SE = 0.003).

The sensitivity of clinical assessment in distinguishing early T stage (T1–2) from advanced T stage (T3–T4) was 73.3% (95%CI: 72.7–73.8%), specificity was 97.7% (95%CI: 97.4–97.9%), accuracy was 82.3% (95%CI: 81.9–82.7%), PPV was 98.1% (95%CI: 97.9–98.3%) and NPV was 68.4% (95%CI: 67.9–68.8%). The sensitivity of clinical assessment in detection of nodal disease (N1–2 stage) was 45.8% (95%CI: 45–46.6%), specificity was 97.3% (95%CI: 97.1–97.5%), accuracy was 78.7% (95%CI: 78.3–79.1%), PPV was 90.5% (95%CI: 89.9–91.1%), and NPV was 76% (95%CI: 75.8–76.3%) (Table 1).

**Table 1:** Characteristics of the study cohort

Factor	Group	Overall
Mean age in years (SD)		68.2 (13.3)
Sex (%)	Male	61,261 (48.8)
	Female	64,212 (51.2)
Race (%)	White	104,181 (83.0)
	American Indian	420 (0.3)
	Asian	3862 (3.1)
	Black	14,840 (11.8)
	Other	1254 (1.0)
	Unknown	916 (0.7)
Charlson Deyo Score (%)	0	85,736 (68.3)
	1	26,835 (21.4)
	2	8264 (6.6)
	3	4638 (3.7)
Insurance (%)	Medicaid	6350 (5.1)
	Medicare	71,609 (57.1)
	Other government	1070 (0.9)
	Private insurance	40,822 (32.5)
	Not insured	3547 (2.8)
	Unknown	2075 (1.7)
Residence area (%)	Metropolitan	104,890 (85.6)
	Urban	15,683 (12.8)
	Rural	1991 (1.6)
Clinical TNM stage (%)	I	59,325 (47.3)
	II	40,135 (32.0)
	III	26,013 (20.7)
Surgical approach (%)	Open	54,579 (46.1)
	Laparoscopic	56,060 (47.3)
	Robotic assisted	7779 (6.6)
Surgery type (%)	Partial colectomy	43,509 (34.7)
	Subtotal colectomy/hemicolectomy	76,077 (60.6)
	Total colectomy	3526 (2.8)
	Total proctocolectomy	666 (0.5)
	Colectomy, NOS	1695 (1.4)
Pathologic TNM stage (%)	0	534 (0.4)
	I	33,937 (28.5)
	II	41,260 (34.7)
	III	40,866 (34.4)
	IV	2361 (2.0)

### Diagnostic Accuracy of Clinical Staging

Overall, the sensitivity of the clinical assessment ranged from 64.3% to 77.2% for the T stage, from 41.6% to 54.5% for the N stage, and from 62.2% to 70.4% for the TNM stage. The specificity ranged from 96.7% to 97.7% for the T stage, from 95.7% to 97.3% for the N stage, and from 99% to 99.3% for the TNM stage. The accuracy ranged between 76.2% and 82.6% for the T stage, between 76.8% and 81.9% for the N stage, and between 74% and 76.7% for the TNM stage (Table 2).

**Table 2:** Summary of the diagnostic accuracy of the clinical T and N stage

Group		Concordance with pathology	Sensitivity (%)	Specificity (%)	Accuracy (%)	Positive predictive value (%)	Negative predictive value (%)
Entire cohort	T stage	0.606	72.7	97.7	81.3	98.3	64.4
	N stage	0.506	50.7	96.9	80.6	89.8	78.4
	TN stage	0.582	67.1	99	83.3	98.5	75.7
Right colon	T stage	0.594	72.3	97.5	80.4	98.4	62.3
	N stage	0.530	54.5	96.5	81.9	89.3	79.8
	TN stage	0.591	70.4	99	84.7	98.6	76.7
Left colon	T stage	0.624	73.3	97.7	82.3	98.1	68.4
	N stage	0.472	45.8	97.3	78.7	90.5	76
	TN stage	0.572	62.2	99.3	81.4	98.3	74

### Under-Staging and Over-Staging

Under-staging of the T stage in clinical assessment was noted in 24.6% of patients in the entire cohort (25.7% in right colon cancer and 22.6% in left colon cancer) whereas over-staging was observed in 3% of patients (1.6% in right colon cancer and 3.9% in left colon cancer) (Tables 3, 4, 5).

**Table 3:** Under-staging and over-staging in the entire cohort

Clinical T stage	Under-staged	Correctly staged	Over-staged
0	127 (90.1%)	14 (9.9%)	0
1	20,419 (49.5%)	19,589 (47.5%)	1199 (2.9%)
2	5507 (29.4%)	12,861 (68.6%)	364 (1.9%)
3	2680 (5.9%)	41,257 (92.1%)	867 (1.9%)
4	0	10,765 (90.5%)	1125 (9.4%)
Total	28,733 (24.6%)	84,486 (72.4%)	3555 (3%)
Clinical N stage	Under-staged	Correctly staged	Over-staged
0	20,525 (21.6%)	74,420 (78.4%)	0
1	2645 (15.3%)	12,473 (72%)	2201 (12.7%)
2	0	5824 (93.3%)	415 (6.6%)
Total	23,170 (19.5%)	92,717 (78.2%)	2616 (2.2%)
Clinical TNM	Under-staged	Correctly staged	Over-staged
I	21,672 (39.6%)	33,035 (60.4%)	0
II	8581 (22.7%)	28,644 (75.8%)	576 (1.5%)
III	0	21,672 (90.1%)	23,86 (9.9%)
Total	30,253 (25.9%)	83,351 (71.5%)	29,62 (2.6%)

**Table 4:** Under-staging and over-staging in right-sided colon cancer

Clinical T stage	Under-staged	Correctly staged	Over-staged
0	72 (94.7%)	4 (5.3%)	0
1	11,033 (55.8%)	8368 (42.3%)	357 (1.8%)
2	2929 (28.4%)	7208 (70%)	161 (1.6%)
3	1371 (5.8%)	21,936 (92.4%)	442 (1.9%)
4	0	5352 (90.5%)	561 (9.5%)
Total	15,405 (25.8%)	42,868 (71.7%)	1521 (2.5%)
Clinical N stage	Under-staged	Correctly staged	Over-staged
0	9645 (20.1%)	38,198 (79.8%)	0
1	1612 (16.9%)	6603 (69.6%)	1275 (13.4%)
2	0	3252 (94.1%)	203 (5.9%)
Total	11,257 (18.5%)	48,053 (79%)	1478 (2.4%)
Clinical TNM	Under-staged	Correctly staged	Over-staged
I	10,760 (39.5%)	16,466 (60.5%)	0
II	4070 (20.9%)	15,031 (77.4%)	311 (1.6%)
III	0	11,769 (89.7%)	1356 (10.3%)
Total	14,830 (25%)	43,266 (72.7%)	1369 (2.3%)

**Table 5:** Under-staging and over-staging in left-sided colon cancer

	Under-staged	Correctly staged	Over-staged
*Clinical T stage*			
0	42 (82.4%)	9 (17.6%)	0
1	6891 (41.1%)	9151 (54.6%)	730 (4.3%)
2	1894 (29.9%)	4282 (67.6%)	159 (2.5%)
3	889 (5.7%)	14,191 (91.9%)	355 (2.2%)
4	0	3885 (89.9%)	435 (10.1%)
Total	9716 (22.6%)	31,518 (73.4%)	1679 (3.9%)
*Clinical N stage*			
0	8505 (23.9%)	27,002 (70.1%)	0
1	737 (12.6%)	4433 (75.8%)	679 (11.6%)
2	0	1931 (92.1%)	165 (7.9%)
Total	9242 (21.2%)	33,366 (76.8%)	844 (1.9%)
*Clinical TNM*			
I	78,33 (37.7%)	12,924 (62.3%)	0
II	3443 (25.8%)	9688 (72.6%)	207 (1.6%)
III	0	7454 (90.8%)	755 (9.2%)
Total	11,176 (26.5%)	30,066 (71.1%)	962 (2.3%)

Under-staging of the N stage in clinical assessment was noted in 19.5% of patients in the entire cohort (18.5% in right colon cancer and 21.2% in left colon cancer), whereas over-staging was observed in 2.2% of patients (2.4% in right colon cancer and 1.9% in left colon cancer) (Tables 3, 4, 5).

Under-staging of the TNM stage in clinical assessment was found in 25.9% of patients in the entire cohort (15% in right colon cancer and 25.5% in left colon cancer), whereas over-staging was noted in 2.6% of patients (2.3% in right colon cancer and 2.3% in left colon cancer) (Tables 3, 4, 5).

Overall, advanced stages were more likely to be correctly staged than early stages. T3 and T4 stages were more often correctly staged than T1–2 stages, and N2 stage was staged correctly more often than N0 and N1 stages. While 90.1% of stage III patients were correctly staged, only 60.4% of stage I patients were correctly staged.

The evaluation of each clinical T and N stage against the pathologic stage is shown in the Appendix. Overall, 12.2%, 30.9%, and 6.4% of patients with clinical T1 rectal cancer were found to have pathologic T2, T3, and T4 stages, respectively. For patients with clinical N0 disease, 15.5% and 6.1% were found to have pathologic N1 and N2 stages, respectively. Furthermore, 19.5% of 54,214 patients with clinical stage I disease had pathologic stage II and 19.6% had pathologic stage III.

## Discussion

The present study found that the clinical staging of colon cancer had a moderate agreement with the final pathologic stage for both the depth of tumor infiltration and nodal involvement. While the clinical assessment was very specific, it had a suboptimal sensitivity in distinguishing between early and advanced T stages and detection of nodal involvement. The diagnostic accuracy of clinical assessment was overall similar between right and left colon cancers.

Perhaps the most important finding of our study was that the clinical assessment was more accurate in detecting advanced T and N stages as less than 3% of patients were clinically over-staged. Therefore, overtreatment with neoadjuvant chemotherapy may be less likely in patients deemed to have advanced colon cancer in clinical preoperative assessment. However, more patients with advanced disease are likely to be undertreated as almost one-quarter of patients with advanced disease were clinically under-staged and thus may not be indicated for neoadjuvant chemotherapy. Therefore, it may be advisable to re-assess patients with early disease in the clinical assessment to ascertain the absence of advanced T or N stage before proceeding to surgery.

The second important finding was that, although the clinical assessment of T and N stages of colon cancer was very specific, it had only a modest sensitivity. The specificity of clinical assessment in differentiating early and advanced T stage of colon cancer exceeded 95%; however, the sensitivity was much lower and ranged from 64.3% to 77.2%. A meta-analysis^8^ reported a 77% sensitivity of CT scanning in the assessment of the depth of colon cancer invasion. Another meta-analysis^10^ reported a higher sensitivity (86%) of CT scan in distinguishing between tumors confined to the colonic wall and tumors invading beyond the muscle layer. A concerning finding in our study was that 23–36% of patients who had locally advanced pT3–4 colon cancer were incorrectly assessed as early T-stage tumors in clinical assessment. This finding implies that up to one-third of patients with locally advanced disease who were indicated for radiation therapy may not receive it since they were clinically under-staged.

Similarly, the clinical assessment of the N stage had a modest sensitivity that was even lower than that of the T stage, ranging between 45.8% and 54.5%. Previous studies^8,10,11^ reported the sensitivity of CT scanning in assessing the N stage to be approximately 70%. Dighe et al.^11^ concluded that CT scanning has a poor ability to identify nodal disease in colon cancer. Conversely, nodal clinical assessment was highly specific as only 2% of patients were over-staged for the N stage, which should minimize overtreatment.

Neoadjuvant chemotherapy may be indicated in locally advanced colon cancer to help decrease the size, lower the stage of the primary tumor, and eradicate micrometastases.^12,13^ Accurate clinical staging is crucial to tailor the use of neoadjuvant chemotherapy to patients with locally advanced disease who may gain benefit from it and avoid overtreatment that may be associated with adverse effects without tangible benefits. The current results imply that under-staging of nodal involvement, which may indicate a role for neoadjuvant therapy, occurred in approximately 20% of patients. This finding implies that one out of five patients with nodal disease, who would otherwise be a candidate for neoadjuvant chemotherapy, was erroneously staged as having no nodal disease.

A previous study from the NCDB assessed the concordance between the clinical and pathologic assessments of colon cancer. Although Dehal et al.^14^ previously reported similar specificity of the clinical assessment of the T and N stages to our study, they reported higher sensitivity for the T stage (80% versus 64.3–77.2% in our study) and the N (60% versus 41.6–54.5%). These differences may be attributable to the different timelines of both studies and differences in the imaging modalities used for the assessment of colon cancer. Moreover, our study included a subgroup analysis of the accuracy of clinical assessment in right versus left colon cancers, which showed a higher concordance between clinical and pathologic T stage in left colon cancers than in right colon cancers. Furthermore, while the previous analysis focused on the T and N stages separately, the present study provided a concordance analysis for the collective TNM stage.

The moderate agreement between clinical and pathologic assessments and high specificity of clinical staging indicates a good diagnostic utility of clinical assessment of colon cancer. Generally, a moderate-to-substantial agreement is considered satisfactory, and a high specificity is necessary to avoid overtreatment by having a few false positive patients who were clinically assessed as having advanced disease but ultimately had early disease on pathologic assessment. However, the modest sensitivity of clinical assessment involves a considerable number of false negative patients who had advanced disease yet were not identified as such in the clinical assessment. This problem may lead to under-treatment of patients with nodal involvement (stage III disease) who may be at an increased risk of having micrometastases and, thus, when clinically under-staged may not have the potential benefit of neoadjuvant chemotherapy to control possible micrometastases. Some authors suggested that using MRI for clinical assessment of colon cancer may confer a greater accuracy than CT scanning. However, this assumption is not necessarily true. A recent study found that MRI had a low sensitivity (43–67%) in detecting T3cd/T4 tumors and a moderate sensitivity in the detection of nodal involvement.^15^ Therefore, there remains an immense need to improve the current methods used to assess nodal affection in colon cancer.

The present study adds to the existing data regarding the diagnostic accuracy of clinical staging of colon cancer by providing data from a large national database. The current study also compared the accuracy of clinical assessment of TN stage between right and left colon cancers. We found that left colon cancers showed higher concordance between clinical and pathologic T stages, yet a lower concordance between clinical and pathologic N stages when compared with right colon cancers. Another strength of the study is the use of different metrics of diagnostic accuracy that included kappa of concordance, sensitivity, specificity, and predictive values. While concordance examined the overall agreement between clinical and pathologic stages, sensitivity assessed the false negatives, and specificity assessed false positives. The excellent specificity of clinical assessment indicates that only a few patients will be erroneously diagnosed in clinical assessment as having an advanced disease.

The present study has some limitations including the inherent weaknesses of database-derived studies such as missing data on the clinical stage of many patients, the possibility of misclassification and inaccuracies, and the retrospective nature of data. Unfortunately, owing to entry limitations of the database, we were unable to determine the imaging method used for clinical assessment, whether CT scanning or MRI, which might have provided useful data by comparing the diagnostic accuracy of both modalities. The findings of our study are consistent with similar analyses of other gastrointestinal cancers using the NCDB.^16^ Therefore, there might be some risk of information bias inherent to the NCDB. The limitations of the current imaging modalities used for staging need to be considered when discussing treatment options with patients with colon cancer, particularly patients with early T and N stages in clinical assessment as 20–25% of them may otherwise have more advanced disease.

## Conclusions

The clinical assessment of T and N stages of colon cancer had good diagnostic accuracy with a moderate concordance with the final pathologic stage. While clinical assessment of T and N stages was highly specific with < 3% of patients being over-staged, it had a modest sensitivity, especially for detection of nodal involvement. Advanced T and N stages were more likely to be correctly staged than were earlier stages. The diagnostic accuracy of clinical assessment of right and left colon cancers was similar, except for a higher sensitivity and accuracy of assessment of nodal involvement in right than left colon cancers.

## Supplementary Information

Below is the link to the electronic supplementary material.

Supplementary file1 (DOCX 17 kb)
