# Supplementary material for: Accuracy of Clinical Staging of Localized Colon Cancer: A National Cancer Database Cohort Analysis
Source: Ann Surg Oncol. 2024 Jul 29;31(10):6461–9. doi: 10.1245/s10434-024-15875-9 (PMC11413029; doi:10.1245/s10434-024-15875-9)
Supplement: Supplementary file 1 — Supplementary file1 (DOCX 17 kb) [file 10434_2024_15875_MOESM1_ESM.docx]

**Breakdown of clinical T-stage**

|  | **Pathologic T0** | **Pathologic T1** | **Pathologic T2** | **Pathologic T3** | **Pathologic T4** | **Total** |
| --- | --- | --- | --- | --- | --- | --- |
| **Clinical T0** | 14 (9.9) | 37 (26.2) | 30 (21.3) | 41 (29.1) | 19 (13.5) | 141 |
| **Clinical T1** | 1199 (2.9) | 19589 (47.5) | 5039 (12.2) | 12742 (30.9) | 2638 (6.4) | 41207 |
| **Clinical T2** | 41 (0.2) | 323 (1.7) | 12861 (68.6) | 4676 (24.9) | 831 (4.4) | 18732 |
| **Clinical T3** | 14 (0.03) | 97 (0.2) | 756 (1.7) | 41257 (92.1) | 2680 (5.9) | 44804 |
| **Clinical T4** | 3 (0.02) | 8 (0.07) | 55 (0.5) | 1059 (8.9) | 10765 (90.5) | 11890 |

****Number (%)***

**Breakdown of clinical N-stage**

|  | **Pathologic N0** | **Pathologic N1** | **Pathologic N2** | **Total** |
| --- | --- | --- | --- | --- |
| **Clinical N0** | 74420 (78.4) | 14738 (15.5) | 5787 (6.1) | 94945 |
| **Clinical N1** | 2201 (12.7) | 12473 (72) | 2645 (15.3) | 17319 |
| **Clinical N2** | 211 (3.4) | 204 (3.3) | 5824 (93.3) | 6239 |

****Number (%)***

**Breakdown of clinical of TNM stage**

|  | **Pathologic TNM I** | **Pathologic TNM II** | **Pathologic TNM III** | **Total** |
| --- | --- | --- | --- | --- |
| **Clinical TNM I** | 33035 (60.9) | 10566 (19.5) | 10613 (19.6) | 54214 |
| **Clinical TNM II** | 576 (1.5) | 28644 (75.8) | 8581 (22.7) | 37801 |
| **Clinical TNM III** | 326 (1.3) | 2060 (8.6) | 21672 (90.1) | 24058 |

****Number (%)***
